# Life after radical cystectomy: A mixed‐methods targeted review of patient‐reported quality of life following bladder removal

**DOI:** 10.1002/bco2.70049

**Published:** 2025-09-10

**Authors:** Ingolf Griebsch, Kristian Juul, Andrew Bottomley, Roya Sherafat‐Kazemzadeh, Jack Pemment, Tori Brooks, Rocco Adiutori, Sonia Bothorel

**Affiliations:** ^1^ Ferring Pharmaceuticals A/S Copenhagen Denmark; ^2^ Bottomley Consulting Group Overijse Belgium; ^3^ MAPI Research Trust Boston USA; ^4^ ICON Plc. Collegeville USA; ^5^ MAPI Research Trust Athens USA; ^6^ MAPI Research Trust Bologna Italy; ^7^ MAPI Research Trust Lyon France

**Keywords:** bladder cancer, mixed methods, patient experience, patient reported outcomes, quality of life, radical cystectomy

## Abstract

**Background:**

Radical cystectomy (RC) is a life‐altering surgery primarily used to treat muscle‐invasive bladder cancer (MIBC) and, occasionally, high‐risk non‐muscle‐invasive bladder cancer (NMIBC). While this procedure can be lifesaving, it often leads to significant changes in quality of life (QOL). This review synthesizes the current quantitative and qualitative literature on QOL outcomes for RC patients, highlighting areas of impact and minimal recovery post RC.

**Methods:**

A targeted literature review was conducted in Medline, searching for studies using qualitative methods to report patient experience and important aspects of QOL outcomes among RC patients between 2013 and 2024. A second search was performed focusing on clinical studies that reported QOLs using quantitative methods. Studies were screened based on study population and type of reported outcomes. Nine qualitative studies were selected to identify important themes related to QOL concepts. There were seven quantitative studies that were selected to extract the results of reported patient outcomes. These results were categorized using the themes identified (Sexual Functioning, Physical Functioning, Emotional Functioning, Work Functioning, Activities of Daily Living and Family‐Social Functioning). Key QOL areas were examined and organized by the severity of impairment and potential for recovery.

**Results:**

Patients experienced disease impact on sexual functioning and physical mobility as well as emotional well‐being, daily living activities, work functioning and social interactions, with the first two domains most profoundly affected by RC. Emotional challenges and dependence on family support were prevalent post RC, with some gradual improvements in the second year. Qualitative findings also underscore the complex emotional and social adjustments patients undergo.

**Conclusion:**

This review highlights the extensive impact of RC on multiple dimensions of QOL, suggesting a critical need for improved patient counselling and long‐term support strategies. The findings highlight the importance of educating patients about the potential changes in QOL when considering treatment options. With shared patient and clinician decision making in specific cases of NMIBC, bladder sparing strategies may be considered, depending on the clinical contexts and patients' individual needs.

## 1 INTRODUCTION

Bladder cancer is the ninth most common cancer worldwide, with 25% of cases diagnosed at later stages of progression.^1^ In 2024, the American Cancer Society estimated 83 190 new cases of bladder cancer and 16 840 related deaths in the United States.^2^ For patients undergoing radical cystectomy (RC), the procedure carries a 2.7% 30‐day mortality rate and a 90‐day mortality rate of 4.9%.^3^ Furthermore, RC is associated with high post‐operative complication rates, ranging from 50 to 88% for low‐grade Clavien complications and from 30 to 42% for high‐grade Clavien complications.^4^, ^5^

Radical cystectomy (RC) permanently changes the way the body stores and eliminates urine. The surgery involves the removal of the bladder, as well as neighbouring lymph nodes, and can also involve the partial removal of the bowel, ovaries, uterus, prostate and part of the vagina. This surgical treatment is currently used with neoadjuvant cisplatin based chemotherapy to treat nonmetastatic muscle invasive bladder cancer (MIBC)^6^ and according to NCCN Clinical Practice Guidelines in Oncology^7^, ^8^ for the initial management of high risk non‐muscle invasive bladder cancer (NMIBC), cystectomy is preferred for Bacillus Calmette‐Guerin (BCG) naïve patients with very high risk features (such as lymphovascular invasion) and BCG unresponsive or intolerant patients.^1^

One important issue for patients undergoing RC is their Quality of life (QOL). QOL refers to the patients' overall well‐being, including physical, emotional, social and functional aspects, while Patient‐Reported Outcomes (PRO) are a measurement of any aspect of a patient's health status that comes directly from the patient. Together, QOL and PRO assessments, when used in clinical practice, may help ensure that cancer care focuses on trying to improve how patients feel and function.

QOL assessments in patients with Non‐Muscle Invasive Bladder Cancer (NMIBC) have historically been underrepresented in research.^9^, ^10^ This is surprising because three‐quarters of new bladder cancer diagnoses are NMIBC.^11^ However, a growing number of studies have examined the QOL of NMIBC patients following RC, and to understand the impact of the procedure on patients, we provide a comprehensive synthesis of both qualitative and quantitative literature. Even though we were primarily interested in studies that had a specific focus on NMIBC due to the underrepresentation in the literature, we acknowledge the limited number of such reports and included all studies on patients with Bladder Cancer as a general diagnosis and one paper with a MIBC sub‐population.

By combining data from both quantitative and qualitative studies, we hope to provide a more holistic view of the impact of RC on QOL, particularly as it pertains to NMIBC. The findings are organized around common themes in QOL research (sexual functioning, physical functioning, emotional functioning, daily living activities, work functioning and family and social functioning), highlighting both the measurable outcomes and the personal, lived experiences of patients.

## 2 METHODOLOGY

We first examined publications of articles that used qualitative methods such as focus groups and interviews to solicit concepts and aspects of health that are considered important by the patients. In this paper, we refer to this part of the review as “qualitative study”. Among the nine qualitative studies reviewed here, there was no differential grouping of patients by type of bladder cancer, but three papers included NMIBC as a distinct group and one paper reported on MIBC patients. However, as there was no focus on any specific surgical method or pathology of bladder cancer, this allowed us to capture all aspects and nuances of health that were important to the patients that underwent the procedure.

Additionally, we then included clinical studies that included PRO questionnaires and other surveys to analyse and report QOL among patients who underwent RC. We retrieved quantitative results from patient experience in investigational and observational groups to understand concepts and changes in patient experience as described in these studies. We summarized the findings in this review as the “quantitative part of the study” and further details are described in the following paragraphs.

### 2.1 Search in databases and retrieval of the articles

The landscape review was performed in two rounds, focusing separately on quantitative publications and then qualitative publications that described patient experience following cystectomy. The search was performed in the Ovid database (Medline).

**Qualitative Studies:** the search strategy was based on SPIDER framework: S (Sample): Patient with urologic cancer who underwent cystectomy (partial or radical) or nephrectomy. PI (Phenomena of Interest): impact of the disease on the patients' life from the patients' perspective. D (Design): Semi‐structured in‐depth interview or focus‐group. E (Evaluation): Patient's experience. R (Research): Qualitative studies or mixed‐method studies with a qualitative element, or survey studies. This search was for the timeframe between 2013 and 2023. The qualitative studies identified key concepts reported by the patient describing their illness and/or treatment experience. These concepts were categorized into themes related to the QOL framework such as physical, emotional and social health and functioning,^12^ which were then used to categorize results from the below quantitative search.

**Quantitative reports:** PICOs model was used for search: P (Patient/Population): Patient with urologic cancer ‐ a broader set of keywords pertaining to malignant neoplasms of the urinary tract and bladder cancer. I (Intervention): all types of clinical trials. C (Comparator): Single‐arm studies. O (Outcome): Patient Reported Outcome (PRO) and QOL. S (Study design): interventional and observational studies (excluding case‐reports). This search was limited to studies between 2014 and June 2024.^2^

Appendix A provides the details of the search algorithms for each of the searches (Table A1 and Table A2).

**FIGURE 1:**
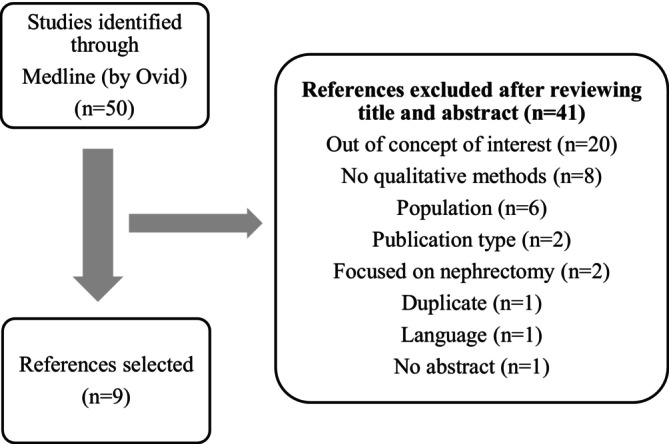
Screening process for qualitative review. This flowchart shows the screening process for choosing qualitative studies that described patient experience and the QOL of patients who underwent RC (without specific discrimination on the method of surgery). From a shortlist of 50 articles retrieved from a preliminary search in Medline, an expert researcher excluded 41 (details depicted in the figure) and 9 were selected for detailed review. A second expert inspected the list and selection criteria and confirmed the list.

**FIGURE 2:**
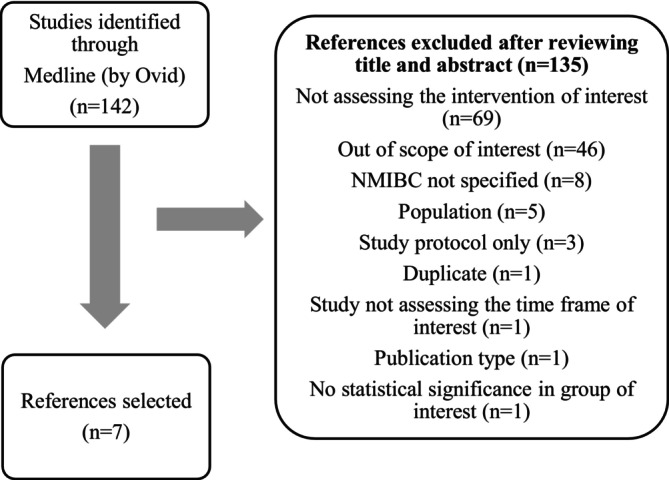
Screening process for quantitative review. This flowchart shows the screening process for choosing quantitative studies that focused on the QOL of RC patients. From a shortlist of 142 articles, 135 were excluded, mainly for being outside of the scope of interest. The screening was performed by 1 expert researcher and reviewed by second experts who confirmed the selections.

The articles retrieved from Medline were further examined by an expert reviewer^3^ to exclude those that were not in scope, were duplicates, or did not use the intended methodology or type of intervention, etc. A second expert reviewer (clinical specialist)^4^ then further inspected the list and finalized the selections for this review: nine for qualitative studies and seven articles for quantitative. Please refer to Figures 1 and 2 for details of screening.

### 2.2 Synthesis of summary results

Study findings within the specific domains are summarized to highlight the nuances of patient experience based on underlying condition (type of cancer), timing of observation and treatment modalities.

## 3 RESULTS

We found nine articles for qualitative studies and seven for quantitative, summarized in Tables 1 and 2, respectively. For quantitative findings, the post RC surgery group is often compared to a comparator group, such as those who had alternate treatment e.g. TURBT, immunotherapy, chemotherapy, or a healthy control group, or in some cases, the scores from the same post RC surgery group are being contrasted at different times points. The comparator has been stated in each case and are summarized for each study in Table 2.

**TABLE 1:** Summary of the studies used in the qualitative review.

Study reference	Patient population	Postoperative period	Data collection method	Patient topic
Yi et al, 2022	BC	Range 1–36 months, median 12 months	Individual interviews	Illness experiences
Rammant et al, 2021	BC	Months since radical cystectomy (mean, [range]): 14 [1–48]	Individual interviews	Supportive care needs
Gupta et al, 2021	BC	All postoperative interviews were conducted at a median of 1 year (interquartile range: 1–2) post radical cystectomy	Individual interviews and a focus group (for postoperative cohort only)	Psychosocial and sexual experiences and concerns
McMullen et al, 2019	BC	Surgeries took place 2.6 years (mean) before study participation (range: 1–6 years) in the first centre, and 2.1 (mean) years before study participation (range: 0–5 years) in the second centre	Focus groups and individual interviews	Needs and challenges before and after RC surgery
Garg et al, 2018	NMIBC	‐	Focus groups	Experiences and care priorities
Cerruto et al, 2014	BC	Patients undergoing radical cystectomy and ileal conduit with a follow‐up from one year up to more than 7 years after RC surgery	Individual interviews	Evolution of the needs and expectations
Mohamed et al, 2014	MIBC	Time since treatment, median (interquartile range in months): 16.2 (27) Time between the diagnosis and the interview, median (interquartile range in months): 17.8 (63)	Individual interviews	Unmet informational and supportive care needs along the illness trajectory
Kowalkowski et al, 2014	NMIBC	‐	Individual interviews	Impact of sexual dysfunction
Fitch et al, 2010	BC	Each participant was interviewed on one occasion 12 to 36 months after his or her surgery	Individual interviews, followed by a focus group	Quality of life after RC surgery

Key:

BC: Bladder cancer group (Including patients with all indications that had radical cystectomy); MIBC: Muscle Invasive Bladder Cancer group; NMIBC: Non Muscle Invasive Bladder Cancer Group.

**TABLE 2:** Summary of studies used in quantitative review.

Rogers et al. (2024)	
Country	UK
Study objective	To describe QOL of patients with newly diagnosed bladder cancer and compare with national cancer and general population.
Study type	Prospective observational cohort study (12‐month)
Study population	Adults 18 + years: (n = 296: 79% male; 21% female) Patients with new diagnosis of bladder cancer (including MIBC and NMIBC)
Design (intervention, comparison groups, timeframe, response rate)	TURBT ± intravesical therapy (238 patients, 80%); radical cystectomy (33 patients, 11%); radiotherapy (18 patients, 6%); palliation (7 patients, 2%); Cancer Quality of Life Survey and Health Survey for England (comparison group)
PRO data collection (schedule of assessment)	3–6 months post‐surgery (post treatment period), 9–12 months post‐surgery (recovery period)
Concepts measured	GLTEQ scores: ‘active’; ‘moderately active’; ‘inactive/sedentary’ EQ‐5D in domains of mobility, self‐care, usual activities, pain/discomfort, anxiety/depression VAS of overall health: scores 0 (worst) –100 (best possible health) EORTC QLQ C30 ‐ domains: overall Global Health, Physical, Role, Emotional, Cognitive and Social function and Fatigue, Pain, Nausea/Vomiting, Dyspnoea, Insomnia, Lack of Appetite, Constipation, Diarrhoea and Financial Issues EORTC QLQ‐NMIBC24; EORTC QLQ‐BLM30 modules on items relevant to both patients with NMIBC and MIBC
PRO Questionnaire(s)	EQ‐5D; EORTC QLQ‐30‐item core; EORTC QLQ‐24‐item NMIBC; EORTC QLQ‐30‐MIBC; EORTC QLQ‐BLM30
Mastroianni et al. (2023a)	
Country	Italy
Study objective	To compare University of Southern California (USC) Institute of Urology pentafecta and trifecta achievement comparing open radical cystectomy (ORC) vs robot‐assisted RC (RARC) with totally intracorporeal urinary diversion (iUD) from a randomized controlled trial (RCT).
Study type	Randomized controlled trial (RCT)
Study population	Robot Assisted Radical Cystectomy (RARC): n = 58 adults (62 ± 10.2 years) n = 44 male (76%); n = 14 female (24%) Open Radical Cystectomy (ORC): n = 58 adults (64 ± 8.4 years) n = 40 male (69%); n = 18 female (31%)
Design (intervention, comparison groups, timeframe, response rate)	ORC (58 patients) against RARC (58 patients)
PRO data collection (schedule of assessment)	1 and 2 years post radical cystectomy
Concepts measured	EORTC QLQ C30 ‐ domains: overall Global Health, Physical, Role, Emotional, Cognitive and Social function and Fatigue, Pain, Nausea/Vomiting, Dyspnoea, Insomnia, Lack of Appetite, Constipation, Diarrhoea and Financial Issues
PRO Questionnaire(s)	EORTC QLQ‐30‐item core
Mastroianni et al. (2023b)	
Country	Italy
Study objective	To identify the most impaired QOL features in patients receiving a radical cystectomy, compared to a healthy population control, as well as patients' recovery after RC surgery, providing high‐level evidence for QOL assessment, in order to minimize the undeniable impact of surgery on daily life.
Study type	Randomized controlled trial (RCT)
Study population	Patients with RC: n = 25 adults (18 < 75 years (72%), 7 ≥ 75 years (28%)) n = 19 male (76%), n = 6 female (24%) Patients with mBCG: n = 25 adults (18 < 75 years (72%), 7 ≥ 75 years (28%)) n = 22 male (88%), n = 3 female (12%)
Design	Patients with BCa (n = 116), who were candidates
(intervention, comparison groups, timeframe, response rate)	for radial cystectomy (RC) with curative intent, against an HC (n = 302)
PRO data collection (schedule of assessment)	Baseline, 6, 12 and 24 months post RC surgery
Concepts measured	EORTC QLQ C30 ‐ domains: overall Global Health, Physical, Role, Emotional, Cognitive and Social function and Fatigue, Pain, Nausea/Vomiting, Dyspnoea, Insomnia, Lack of Appetite, Constipation, Diarrhoea and Financial Issues
PRO Questionnaire(s)	EORTC QLQ − 30‐item core
Fu et al. (2021)	
Country	China
Study objective	To compare the efficacy and complications of bilateral cutaneous ureterostomy with a single sub umbilical stoma to those of cutaneous ureterostomy with two stomas and an ileal conduit as a means of urinary diversion after radical cystectomy.
Study type	Observational study with retrospective data analysis
Study population	Single stoma patients: n = 27 adults (71.9 ± 9.4 years) n = 24 male (85.2%); n = 3 female (14.8%) Two stomas' patients: n = 45 adults (70.1 ± 10.1 years) n = 41 male (91.1%); n = 4 female (8.9%) Ileal conduit patients: n = 36 adults (63.6 ± 7.3 years) n = 31 male (86.1%); n = 5 female (13.9%)
Design (intervention, comparison groups, timeframe, response rate)	Patients (n = 108) who received bilateral cutaneous ureterostomy with a single sub umbilical stoma (ureterostomy with a single stoma group), cutaneous ureterostomy with two stomas (ureterostomy with two stomas group), or an ileal conduit (ileal conduit group) after radical cystectomy.
PRO data collection (schedule of assessment)	One time <1 year after RC surgery
Concepts measured	Urinary, bowel and sexual function
PRO Questionnaire(s)	Bladder Cancer Index (BCI)
Tsai et al. (2021)	
Country	Taiwan
Study objective	The aims of this study were to explore the factors associated with QOL changes after controlling for potential confounding comorbidities and to explore the long‐term dynamic changes in QOL among bladder cancer patients who did and did not receive a cystectomy.
Study type	Observational study
Study population	All patients: n = 343 adults (67.0 ± 10.5 years) n = 238 male; n = 105 female Patients with cystectomy: n = 52 adults (67.7 ± 9.4 years) n = 46 male; n = 17 female Patients without cystectomy: n = 291 adults (67.0 ± 11.8 years) n = 192 male; n = 88 female
Design (intervention, comparison groups, timeframe, response rate)	Long‐term dynamic changes in QOL among bladder cancer patients who did and did not receive a cystectomy Fifty‐two (15%) patients underwent radical cystectomy (radical cystectomy + neobladder, 19; radical cystectomy + ileal conduit, 25; radical cystectomy + ileal reservoir with the Mitrofanoff procedure, 4; radical cystectomy alone, 4)
PRO data collection (schedule of assessment)	At visits and follow‐ups to 5 + years post RC surgery
Concepts measured	Physical, psychological, social relationship and environment domains
PRO Questionnaire(s)	World Health Organization Quality of Life assessment instrument (WHOQOL‐BREF)
Catto et al. (2021)	
Country	UK
Study objective	To understand whether a randomized trial comparing intravesical maintenance Bacillus Calmette‐Guerin (mBCG) and radical cystectomy (RC) for high‐grade non‐muscle invasive bladder cancer (HRNMIBC) was possible.
Study type	Two‐arm randomized controlled trial (RCT)
Study population	Patients with RC: n = 25 adults (18 < 75 years (72%), 7 ≥ 75 years (28%)) n = 19 male (76%), n = 6 female (24%) Patients with mBCG: n = 25 adults (18 < 75 years (72%), 7 ≥ 75 years (28%)) n = 22 male (88%), n = 3 female (12%)
Design (intervention, comparison groups, timeframe, response rate)	Comparison of paring intravesical maintenance Bacillus Calmette‐Guerin (mBCG) (n = 25) against radical cystectomy (RC) (n = 25) treatment for high‐grade non‐muscle invasive bladder cancer (HRNMIBC)
PRO data collection (schedule of assessment)	3, 6 and 12 months post RC surgery
Concepts measured	EQ‐5D in domains of mobility, self‐care, usual activities, pain/discomfort, anxiety/depression. EORTC QLQ C30 ‐ domains: overall Global Health, Physical, Role, Emotional, Cognitive and Social function and Fatigue, Pain, Nausea/Vomiting, Dyspnoea, Insomnia, Lack of Appetite, Constipation, Diarrhoea and Financial Issues. EORTC QLQ‐NMIBC24; EORTC QLQ‐BLM30 modules on items relevant to both patients with NMIBC and MIBC.
PRO Questionnaire(s)	EQ‐5D‐3L EORTC QLQ‐C30 EORTC NMIBC‐24 EORTC QLQ‐BLM30
Jung et al. (2020)	
Country	USA
Study objective	To examine the effect of non‐muscle‐invasive bladder cancer (NMIBC) diagnosis and treatment on survivors' quality of life (QOL).
Study type	Population‐based cross‐sectional study
Study population	n = 376 adults (68.3 ± 9.2 years) n = 272 male (72.3%), n = 104 female (27.7%)
Design (intervention, comparison groups, timeframe, response rate)	Previous treatments: TURBT only: n = 88 (24.7%) TURBT + Chemotherapy: n = 94 (26.3%) TURBT + Immunotherapy: n = 123 (34.5%) TURBT + chemotherapy + immunotherapy: n = 31 (8.7%) Cystectomy: n = 21 (5.9%)
PRO data collection (schedule of assessment)	One time for patients 1–6 years post diagnosis
Concepts measured	EORTC QLQ C30 ‐ domains: overall Global Health, Physical, Role, Emotional, Cognitive and Social function and Fatigue, Pain, Nausea/Vomiting, Dyspnoea, Insomnia, Lack of Appetite, Constipation, Diarrhoea and Financial Issues. EORTC QLQ‐NMIBC24; EORTC QLQ‐BLM30 modules on items relevant to both patients with NMIBC.
PRO Questionnaire(s)	EORTC QLQ‐C30 EORTC NMIBC‐24

Key:

mBCG; Bacillus Calmette‐Guerin; MIBC: Muscle Invasive Bladder Cancer; NMIBC: Non‐muscle Invasive Bladder Cancer; PRO: Patient Reported Outcome; QOL: Quality of Life; RC; radical cystectomy; TURBT: TransUrethral Resection of Bladder Tumour.

PRO Questionnaires.

BCI: Bladder Cancer Index; EQ‐5D: EuroQOL five Dimensions (3 Level /5 Level/VAS: Visual Analogue Scale); EORTC QLQ C30: European Organization for Research and Treatment of Cancer quality of life questionnaire; EORTC NMIBC‐24: European Organization for Research and Treatment of Cancer quality of life questionnaire non‐muscle‐invasive bladder cancer; EORTC QLQ‐BLM30: European Organization for Research and Treatment of Cancer Quality of Life Questionnaire ‐ Muscle Invasive Bladder Cancer; GLTPEQ ‐ Godin Leisure‐Time Physical Activity Questionnaire; WHOQOL‐BREF: World Health Organization Quality of Life assessment instrument.

The qualitative findings were arranged into the key concept categories and domains describing QOL. These domains directly reflect patients' perspectives that complement the quantitative data. The results are organized by broad QOL domains and in order of impact severity, with the most severely affected domains showing minimal evidence of recovery based on quantitative reports. Qualitative QOL common issues within each functional area are shown in Table 3, whereas key quantitative results are shown in Tables 4, 5 and 6.

**TABLE 3:** Summary of topics discussed within the nine articles of the qualitative literature review.

Impact on QOL	Topics
Family and Social Functioning	Depending on others (decreasing with time)
	Role changing/Loss of equity/Stress on partner
	Loss of intimacy because of sexual functioning/body image (affected by age)
	Challenging to establish new sexual relationships
	Social isolation because of emotional impact and physical function (decreasing with time)
Sexual Functioning	General sexual dysfunction due to symptoms (e.g. erectile dysfunction)
	Libido and pain during intercourse
	Priority related with age
Work functioning	Unable to work
Emotional Functioning	Fear/Anxiety/Worry due to cancer diagnosis
	Depression
	Low confidence/self‐esteem because of body image
	Bother/Embarrassment with incontinence/voiding
	Disappointment with family members/friends
	Concerned/Worried with RC impact on sexual functioning/body image (preoperative)
	Regretting the past
	Feeling a burden to others
	Worried about the future
	Feeling constrained/Sense of loss/Sense of shame due to ADL and physical function changes
	Fear of sexual activity
	Uncomfortable/Embarrassment with sexual dysfunction
	Bother/Embarrassment with sexual dysfunction
	Not feeling normal (after RC surgery)
	Confusion due to physical function changes
Daily Living Activities	Need of immediate access to toilet affects ADL, including travelling
	Exercising
	Getting dressed
	General daily tasks (reduce with time)
	Sleeping habits
	Daily tasks due to pain and fatigue
	Eating habits
	Toileting/self‐care
Physical Functioning	Urinary function
	Difficult sleeping
	Loss of stamina/Fatigue
	Cognitive function
	Physical recovering (urinary tract infections)

This table summarizes the impact of radical cystectomy on different issues within each QOL area.

**TABLE 4:** Summary of quantitative results for sexual and physical functioning.

Study	Sexual functioning	Physical functioning
Rogers et al. (2024)	**Change: EORTC QLQ‐C30** (Bladder cancer specific issue): Sexual function mean score^2^: RC‐B: 24.7, RC‐P: 15.1* **Change EORTC QLQ‐C30** (Bladder cancer specific issue): Male sexual problems mean score^3^: RC‐B: 35.8, RC‐P: 66.7* **Change: EORTC QLQ‐C30** (Bladder cancer specific issue): Male sexual problems mean score^3^: RC‐B: 35.8, RC‐R: 75.8**	**Change: EORTC QLQ‐C30** (Bladder cancer specific issue): Physical function mean score^1^: RC‐B: 84.1, RC‐P: 71.0*
Mastroianni et al. (2023b)	NA	**EORTC QLQ‐C30**: At 6**, 12* and 24 months*, the RC group reported worse physical functioning scores than the healthy population control group.
Fu et al. (2023)	**BCI:** All patients reported an inactive sex life and no sexual worries. However, Fu et al. state that the sexual function domain was invalid as all patients reported no sex life or sexual troubles.	**NA**
Catto et al. (2020)	NA	**EORTC QLQ‐C30**: RC patients experienced a drop in physical functioning at 3 months, but this score recovered to baseline between 6 and 12 months.
Jung et al. (2020)	**EORTC QLQ‐NMIBC 24**: Sexual function mean score^2^: RC 15.8 vs alternative treatments (TURBT only [29.6]; TURBT + chemo [27.0]; TURBT + immuno [34.8]; TURBT + chemo + immuno [26.5])* **EORTC QLQ‐NMIBC 24**: Sexual intimacy mean score^4^: RC 50.0 vs alternative treatments (TURBT only [26.8]; TURBT + chemo [27.6]; TURBT + immuno [23.9]; TURBT + chemo + immuno [29.8])* **EORTC QLQ‐NMIBC 24**: Sexual enjoyment mean score^5^: RC 26.3 vs alternative treatments (TURBT only [48.9]; TURBT + chemo [46.7]; TURBT + immuno [52.9]; TURBT + chemo + immuno [42.9])* **EORTC QLQ‐NMIBC 24**: Male sexual enjoyment mean score^6^: RC 66.7 vs alternative treatments (TURBT only [52.3]; TURBT + chemo [48.7]; TURBT + immuno [41.1]; TURBT + chemo + immuno [57.9])*	NA

KEY:

* p‐Value<0.05; ** p‐Value<0.005; *** p‐Value<0.001.

B: Baseline; P: Post‐Treatment = data collected at 3, 6, 9, 12 month timepoints after RC surgery; R: Recovery = 12‐months after RC surgery.

RC: Radical Cystectomy; TURBT: TransUrethral Resection of Bladder Tumour.

Bladder Cancer Index (BCI; European Organization for Research and Treatment of Cancer quality of life questionnaire (EORTC QLQ) ‐ 30‐item core (C30); EORTC QLQ 24‐item non‐muscle‐invasive bladder cancer (EORTC QLQ NMIBC24); EORTC QLQ 30‐item muscle‐invasive bladder cancer (EORTC QLQ BLM30).

In the Mastroianni et al. (2023b) study, the RC group was made up of both patients that had open radical cystectomy (ORC) and robot assisted cystectomy (RAC).

^1^ :Higher score indicates better physical function

^2^ :Higher score indicates better sexual function

^3^ :Higher score indicates worse male sexual function

^4^ :Higher score indicates lower sexual intimacy.

^5^ :Higher score indicates greater sexual enjoyment

^6^ :Higher score indicates lower male sexual enjoyment.

**TABLE 5:** Summary of quantitative results for emotional functioning and daily living activities.

Study	Emotional functioning	Daily living activities
Rogers et al. (2024)	**Equation 5 D:** Proportion reporting anxiety at B: RC: 56%, TURBT: 36%* **EORTC QLQ‐C30:** Future worries mean score^ǂ^ at B: RC: 39.2, TURBT: 29.4** **Change EORTC QLQ‐C30 (Bladder cancer specific issue)**: Future worries mean score^ǂ^ RC‐B: 39.2, RC‐R: 24.8**	**Change Equation 5D**: Proportion reporting mobility issues RC‐B: 32%, RC‐P: 48%* **Change Equation 5D**: Proportion reporting issues with usual activities RC‐B: 47%, RC‐P: 78%*
Mastroianni et al. (2023b)	**EORTC QLQ‐C30**: At 6 months**, the RC group reported worse emotional functioning scores than the healthy population control group. At 12 months*, the ORC patients (in contrast to the RAC patients) in the RC group reported worse emotional functioning scores than the healthy population control group.	NA
Tsai et al. (2021)	NA	**WHOQOL‐BREF:** NMIBC RC patients reported lower scores for activities of daily living compared to those that had BST. This difference occurred mainly in the first 40 months following surgery. Scoring was not available in publication.
Catto, et al. (2021)	**EORTC QLQ‐BLM30 and EORTC QLQ‐C30:** Future worries scores were merged to show that **t**he RC experienced a decrease in future worries from the start of recovery (3 months) to the end of recovery (12 months). Scoring not available in publication.	NA

KEY:

* * p‐Value<0.05; ** p‐Value<0.005; ** p‐Value<0.001.

B: Baseline; P: Post‐Treatment = data collected at 3, 6, 9, 12 months timepoints after RC surgery; R: Recovery = 12‐month after RC surgery.

RC: Radical Cystectomy; TURBT: TransUrethral Resection of Bladder Tumour; ORC: Open Radical Cystectomy; RAC: Robot‐Assisted Cystectomy.

EuroQOL five Dimensions (EQ‐5D); Visual Analogue Scale (VAS); European Organisation for Research and Treatment of Cancer quality of life questionnaire (EORTC QLQ) ‐ 30‐item core (C30); EORTC QLQ 24‐item non‐muscle‐invasive bladder cancer (EORTC QLQ NMIBC24); EORTC QLQ 30‐item muscle‐invasive bladder cancer (EORTC QLQ BLM30).

In the Mastroianni et al. (2023b) study, the RC group was made up of both patients that had open radical cystectomy (ORC) and robot‐assisted cystectomy (RAC).

ǂ: Higher score indicates more future worries.

**TABLE 6:** Summary of quantitative results for work functioning and family and social functioning.

Study	Work functioning	Family and social functioning
Mastroianni et al. (2023b)	**EORTC QLQ‐C30**: At 6**, 12** and 24 months, the RC group reported worse financial difficulty scores than the healthy population control group.	**EORTC QLQ‐C30**: At 6** and 12* months, the RC group reported worse social functioning scores than the healthy population control group. At 24 months*, the RAC patients (in contrast to the ORC cystectomy patients) in the RC group reported worse social functioning scores than the healthy population control group.
Fu et al. (2021)	Ostomy‐related costs (median value): SS (298.5), TS (405.4) and IC (305)* Study did not provide currency or financial indicators.	NA
Tsai, et al. (2021)	NA	**WHOQOL‐BREF:** NMIBC RC patients reported worse social domain scores compared to those that had BST. This difference occurred mainly in the first 40 months following surgery. Scoring was not available in publication.

KEY:

* * p‐Value<0.05; ** p‐Value<0.005; ** p‐Value<0.001.

BST: Bladder Sparing Therapy; RC: Radical Cystectomy; SS: Single Stoma; TS: Two Stomas; IC: Ileal Conduit.

European Organization for Research and Treatment of Cancer quality of life questionnaire (EORTC QLQ) ‐ 30‐item core (C30); EORTC QLQ 24‐item non‐muscle‐invasive bladder cancer (EORTC QLQ NMIBC24); EORTC QLQ 30‐item muscle‐invasive bladder cancer (EORTC QLQ BLM30).

In the Mastroianni et al. (2023b) study, the RC group was made up of both patients that had open radical cystectomy (ORC) and robot assisted cystectomy (RAC).

### 3.1 Sexual functioning

The quantitative studies show a marked impact of RC on sexual function. Jung et al.^9^ posted QOL surveys (EORTC QLQ‐C30 and EORTC NMIBC‐24) to 2000 NMIBC patients, with a mean time since diagnosis of 3.4 years. Analysis of 376 questionnaires that were returned (out of 2000) showed that compared to patients who did not have a cystectomy and received alternative treatments (TURBT, immunotherapy, chemotherapy, etc.), patients who underwent a RC (5.6%) reported the highest level of discomfort with sexual intimacy and significantly worse scores in almost all domains of sexual function (measured by EORTC QLQ‐NMIBC24). Rogers et al.^13^ found that sexual functioning and male sexual problems were worse than baseline for both the post RC treatment period (3–12 months after RC surgery) and the recovery period (12 months after RC surgery). Catto et al.^14^ found that there was no improvement in reported sexual function between 3 and 12 months post‐RC surgery.

The most commonly reported issue related to sexual functioning was erectile dysfunction, a specific form of sexual dysfunction associated with treatment‐related symptoms. Yi et al.^15^ stated the increase in sexual dysfunction had been accepted “calmly”, but Gupta et al.^16^ received a response from a cystectomy patient stressing the need for open and honest counselling on sexual matters before the operation. Mohamed et al.^17^ emphasized this point further, noting that only 20% of the patients in their study reported that their physicians mentioned possible changes in sexual function when discussing treatment options.

Only one study^16^ examined sexual functioning in relation to age, and reported two contrasting perspectives. A 68‐year‐old patient was not particularly concerned about sexual intimacy and admitted that it may have been more important if they were younger. In contrast, another patient emphasized, “Even though you get older, you still want that intimacy, so I think it's important.” Sexual function was also assessed by Fu et al.^18^ among 108 participating patients in their study from China (mean age 70). They found that “all patients” in their study indicated that they had an inactive sex life and reported no sexual worries, hence reported no sexual troubles after RC surgery.

### 3.2 Physical functioning

Rogers et al.^13^ discovered that physical functioning was worse during the post RC treatment follow‐up (surveys collected at 3, 6, 9 and 12 month time points) than at baseline (84.1 at baseline vs 71.0 post RC treatment, p = 0.002). Similarly, Mastroianni et al.^4^, ^5^ found that physical functioning was impaired in contrast to a healthy population control group, after 6 (p < 0.001), 12 (p < 0.04) and 24 months (p < 0.045) timepoints following cystectomy. Catto et al.^14^ found that RC patients had a drop in physical functioning at 3 months, but it recovered to baseline between 6 and 12 months.

The most common issue reported from the qualitative review with physical functioning was urinary issues. In Fitch et al.,^19^ patients complained of urinary incontinence or leakage from the stoma. These complaints were further aggravated by the fact that the patients felt the healthcare professionals had not properly instructed them on what to do in the event of leakage. Mohamed et al.^17^ found that 43.33% of patients experienced difficulties with urinary function, such as lack of urine control and leakage. The same study also found that patients reported a lack of adequate training on the use of stomal appliances and catheters.

### 3.3 Emotional functioning

Mastroianni et al.^4^, ^5^ found that six months following cystectomy emotional functioning was impaired by contrast to a healthy control group (p < 0.001). These authors also found at the mid‐term recovery (12 months of follow‐up) patients that underwent open RC had a higher deterioration in emotional functioning compared to the healthy control group (p = 0.03). Rogers et al.^13^ found that 56% of the RC group reported anxiety at baseline, compared to 36% of participants in the TURBT group (p = 0.03) and they also found at baseline that the RC group reported a greater number of future worries compared to the TURBT group (p = 0.005). They also found, however, that the mean future worries score decreased in the RC group from baseline to recovery (p = <0.001). Catto et al.^14^ also found a decrease in future worries score from their RC group at 3 months to 12 months from baseline; however, the raw data is not available.

There were numerous aspects to emotional functioning that were identified in the qualitative publications, seen in interviews and focus groups. Most of the studies found that patients experienced an impact on emotions, such as fear, anxiety and worry due to either the cancer diagnosis or RC. Garg et al.,^9^ reported that one patient “would go through almost anything instead of removal of [their] bladder.” Fitch et al.,^19^ noted that patients wanted the healthcare professionals involved in their treatment to be clear and consistent with information about bladder cancer and treatment plans, and be provided this information in a timely fashion to avoid anxiety and confusion. Rammant et al.^20^ noted that some patients talked to a psychologist after RC surgery, notably about six months after RC, due to emotional difficulties. Other emotional issues experienced by patients that underwent RC were regretting the past, feeling a burden to others, feeling a sense of shame,^15^ and not feeling normal after RC surgery.^17^

### 3.4 Daily living activities

In a quantitative study by Rogers et al.,^12^ the EQ‐5D‐5L and EORTC QLQ‐C30 questionnaires were used to assess patients during the post‐RC treatment period following cystectomy (37 responses in the first 6 months, 10 responses in the second six months). From the EQ‐5D responses, there were more problems with mobility from baseline (32% at baseline vs 48% post RC treatment, p = 0.02) and more problems with carrying out usual activities (47% at baseline vs 78% post RC treatment, p = 0.004). Tsai et al.^21^ found using the WHOQOL‐BREF questionnaire that NMIBC RC, when compared to BST (bladder sparing therapies) patients, reported lower scores for activities of daily living within the first 40 months following surgery.

Qualitative studies provided more context for daily limitations such as getting dressed and changes to sleeping habits,^17^ and changes to eating habits.^15^

### 3.5 Work functioning

Another pivotal issue to surface from our review of the quantitative literature was the financial hardship faced by cystectomy patients. Jung et al.^10^ noted that NMIBC patients in general endure high costs due to bladder cancer care and surveillance. Using the EORTC QLQ‐30, Mastroianni et al.^4^, ^5^ reported that at six, twelve and twenty‐four months post RC surgery time points, cystectomy patients reported more financial difficulties when compared to the healthy population control group (p < 0.003).

In China, Fu et al.^18^ reported that bladder cancer patients faced high costs, mainly because of ostomy‐related expenses, chemotherapy and costs related to routine treatment. They also reported that ostomy costs for single stoma patients are significantly lower than those of two‐stoma patients, and single‐stoma patients are less likely to experience retrograde infection, further lowering the cost of treatment needs.

Only three of the nine qualitative papers covered work functioning. In Yi et al.^15^ one patient stated that they felt forced to leave her employment because of the cystectomy, and she felt like her body was broken. Another patient reported that the operation had left them only able to spend two or three hours in a work meeting before they felt uncomfortable. McMullen et al.,^22^ reported that one cystectomy patient had underestimated the time required to return to work after adjuvant chemotherapy. This patient had initially expected to return to work in two weeks, but the actual recovery period was two months.

### 3.6 Family and social functioning

Social functioning was significantly impacted in the first‐year post RC surgery, with gradual improvements seen by the second year.^4^, ^5^ Tsai et al.^21^ found using the WHOQOL‐BREF questionnaire that NMIBC RC patients, when compared to BST patients, reported an overall decline in the social domain score within the first 40 months following surgery.

Qualitative data revealed that patients initially relied heavily on family members for managing their urostomy, though this dependence lessened over time.^23^ Some patients expressed concerns about forming new intimate relationships due to the presence of a urostomy bag, though others demonstrated a pragmatic approach, accepting their condition and moving forward.^16^ Social isolation caused by the emotional impact and stymied physical function was also only reported in two studies. Patients admitted to going through “the long journey” alone and felt helpless because their life had changed completely.^17^ Cerruto et al.,^23^ asked patients to report on their QOL at multiple time points after their surgery. At five years, patients had become less dependent on their partners, particularly in the management of the urinary stoma, and stated that friendships and social interaction “are enough” for them. At seven years, the recovery of social relations had improved their lives, and they felt that dependence was less of a problem.

## 4 DISCUSSION

When a patient undergoes a radical cystectomy, their QOL is immediately and drastically altered. Their sexual functioning is likely to be impaired, and there appears to be no evidence in the QOL literature of a full recovery. With more research, it might be possible to find data that show patients reporting a recovery of sex functioning.

While the studies show sexual functioning is impaired, there is mixed data on the impact of a subsequently diminished or absent sex life following RC. Fu et al.^18^ reported that all patients answered in the Bladder Cancer Index (BCI) questionnaire that they had an inactive sex life and reported no sexual worries, however this study is from China and the researchers commented that in this culturally conservative context, the elderly (mean age was 70 years old) may have lower sexual demand. Age could be an important factor as one of the participants in Gupta et al.^16^ mentioned that sexual intimacy would matter more if they were younger, but another patient stated even though people get older, sexual intimacy was still important. This shows the nuances related to QOL across different ages and cultures and also underlines the importance of recommendations in the selection of questionnaires used with regards to cultural adaptation and validation.^24^, ^25^, ^26^

Physical functioning is impacted by cancer and by RC. Over the first year, it was found that patients reported a diminished state of physical functioning compared to baseline.^13^ This means that following the surgery, patients felt that their physical condition continued to decline. We found no quantitative studies that reported the physical functioning of RC patients after a period of 12 months, and this could indicate a space for further research. From the nine studies in our qualitative review, we found the most common physical and medical problems that afflicted patients post radical cystectomy were hernias, urinary tract infections or stoma obstructions, challenges to bowel function, pain and fatigue and incontinence. In addition, patients also experienced infections, haematuria, erectile dysfunction, weight gain, vaginal dryness, hernias and lymphedema. These afflictions were either caused or exacerbated by RC, in addition to the patient's somatic responses to cancer and the potential side effects of other treatments.

Quantitative data revealed at baseline the RC group experienced more anxiety and future worries than the TURBT group, but future worries decreased in scores after RC,^13^, ^14^ which seems to indicate a degree of habituation over time, following surgery. The main reasons for the experience of anxiety and fear seemed to be a lack of good, timely communication with the patient's healthcare team regarding treatment plans and what to expect and consequences of illness and treatment options. It would be encouraging to see more research on emotional functioning from longer post‐RC treatment periods.

The impact of RC on daily living activities is significant. We found that daily living activities appeared to decrease the first year after RC, with patients reporting worse mobility and being hindered in their usual day‐to‐day activities from their baseline levels. Inconveniences to daily living activities following RC reflect the way that a patient needs to adapt to their new life. More quantitative data is needed to see if patients' daily living activities would improve from baseline to one year onwards, as such long‐term data was lacking.

A growing number of studies have explored the financial implications of RC. From a quantitative analysis, Jung et al.^10^ reported that NMIBC patients who had a RC had the highest cost to the healthcare system per patient, from diagnosis to death among all cancer types. Even after two years, RC patients were reporting financial difficulties in contrast to a healthy population control group.^4^, ^5^ From a qualitative analysis, patients reported underestimating when they could return to work^22^ and the RC procedure meant they could no longer spend as much time in work meetings.^15^ If patients are held responsible for covering the majority or entirety of their healthcare costs, they are likely to face significant financial strain, potentially leading to a depletion of savings or substantial debt to creditors. The ability to work while receiving cancer therapy and recovering from RC surgery is also limited, which reduces the possibility of patients raising capital to fund their own treatment.

Even though we see a deterioration of social functioning in the first 6–12 months after RC, some quantitative studies reported an improvement in the second year.^4^, ^5^ Some patients reported that they initially became dependent on their partner or family for help with the urinary stoma, but this dependence became less of an issue over time.^23^

The existing literature therefore strongly supports that there is substantial evidence indicating that patients with bladder cancer who underwent RC often have significantly impaired QOL for many months after RC surgery; this finding is supported by the exhaustive Winters et al.^27^ study that found both diminished mental and physical health in 166 RC patients, 14.6 months after diagnosis compared to propensity matched non cancer control patients. In addition, Garg et al.^9^ highlighted the profound psychological and practical concerns associated with this procedure specific to NMIBC patients. One participant recounted the tragic experience of an individual in their community who died by suicide following radical cystectomy, attributed to a markedly diminished QOL. Another participant expressed apprehension about how living with an ostomy significantly disrupted their livelihood, underscoring the deep‐seated fears and challenges that patients associate with this intervention.

Given the well‐documented decrease in QOL following RC and patients' real and often justified fears regarding aspects of recovery, which can be minimal or nonexistent, delaying the surgery becomes an important consideration, even if it does eventually become necessary for survival. A recent study by Taylor et al.^28^ looked at a cohort of 578 BCG‐unresponsive NMIBC patients where some underwent upfront RC (28%) and the remaining patients underwent bladder sparing treatment (72%), and at a median follow‐up of 50 months (20–69 months), there was no statistically significant difference in clinical endpoints (i.e. metastasis‐free survival, cancer‐specific survival, or overall survival between groups). These data suggest that at least one course of bladder‐sparing treatment is safe in the intermediate term with low rates of metastasis and death from bladder cancer, further supporting the window of opportunity in the NMIBC patient population.

An informed and considered trial of bladder sparing therapy in the NMIBC patient population would at least temporarily preserve QOL in vital ways. The sexual and physical decline due to RC could at least be temporarily avoided, as could the post‐RC surgical challenges to emotional health, mobility and an inability to perform day‐to‐day activities. In addition, Catto et al.^14^ concluded that their data did not support RC as the standard of care for all patients.

Our review is not without limitations. This targeted review was designed to provide a structured overview of the landscape of studies using RC and QOL, not to assess the relative efficacy or quality of evidence. As such, we did not undertake a formal risk of bias assessment, which is more appropriate for systematic reviews with meta‐analysis or decision‐making objectives. The studies identified span a wide range of designs and contexts (e.g., RCTs, observational studies, etc.), for which a single RoB tool would not be uniformly applicable. Rather than applying inappropriate or overly generalized assessments, we chose to present key design characteristics and limitations narratively. However, we have transparently reported and described study‐level limitations narratively in the discussion, allowing readers to interpret findings with appropriate caution.

Among the nine qualitative studies reviewed here, there was no differential grouping of patients by type of bladder cancer; three papers included NMIBC as a distinct group, and only one paper reported on MIBC patients. This is a limitation for understanding the full details on patient experience before and after RC surgery and remains an area for future research to enable informed recommendations in terms of timing of surgery in each specific group. This review focused strictly on QOL outcomes; however, it would be interesting for further research to include oncologic outcomes to discover any potential relationship. Also, when reporting financial difficulties, it should be noted only one question from the EORTC QLQ‐30 questionnaire addresses financial difficulties experienced by the cancer patient. This limits the ability for a robust financial analysis. The heterogeneity in study designs, follow‐up periods and QOL measurement tools across the included studies limits direct comparability and may introduce bias. Also, it must be noted that several studies had small sample sizes, particularly in the quantitative studies, and this may affect the generalizability of findings. We also recognize that cultural factors in some studies (e.g., differences in sexual health reporting) may not be broadly applicable.

In conclusion, our mixed‐methods approach provides a comprehensive analysis by capturing both quantifiable outcomes and the nuanced experiences of patients, making it a valuable resource for clinicians, researchers and policymakers focused on enhancing patient‐centered care. Notably, our review highlights the impact of RC on a patient's QOL, which needs careful consideration for patient and clinician alike.

## CONFLICT OF INTEREST STATEMENT

Since the initial planning of the work, **Ingolf Griebsch** remains employed by Ferring Pharmaceuticals. In the past 36 months, he received grants/contracts from Boehringer Ingelheim until September 2023 and from Ferring Pharmaceuticals from October 2023 to the present. Since the initial planning of the work, **Kristian Juul** remains employed by Ferring Pharmaceuticals. Nothing to declare in the past 36 months. Since the initial planning of the work, **Andrew Bottomley** remains employed by Bottomley Consultants. In the past 36 months, Bottomley Consulting Group collaborates with a number of pharmaceutical companies that fund research activities. Since the initial planning of the work, **Roya Sherafat‐Kazemzadeh**, **Tori Brooks**, **Rocco Adiutori**, and **Sonia Botherel** were employed by MAPI Research Trust. Nothing to declare in the past 36 months. Since the initial planning of the work, **Jack Pemment** remains employed by ICON Plc. Nothing to declare in the past 36 months.

## Footnotes

^1^
NCCN guidelines for low or intermediate risk NMIBC patients are to involve bladder sparing strategies or surveillance, and high risk BCG naïve patients with not very high risk features it is recommended to undergo bladder sparing therapies.

^2^
The qualitative search was started in 2023 and so has earlier start and finish dates than the quantitative search, which was started in 2024. In search covered a period of ten years.

^3^
For the quantitative review, the first expert reviewer was Tori Brooks, and for the qualitative review, Rocco Adiutori.

^4^
For both the quantitative and qualitative reviews, the second expert reviewers were Ingolf Griebsch and Kristian Juul.

## Appendix A I

Table A1 and Table A2 detail the search strategies for the quantitative and qualitative studies, respectively.

**TABLE A1:** Search strategy for the retrieval of articles for quantitative studies.

Search #	Algorithm
1. Patient population terms	exp Urologic Neoplasms/or (cancer, urinary tract or cancer, urologic or cancer, urological or “cancer of urinary tract” or “cancer of the urinary tract” or cancers, urinary tract or cancers, urologic or cancers, urological or neoplasm, urinary tract or neoplasm, urologic or neoplasm, urological or neoplasms, urinary tract or neoplasms, urologic or neoplasms, urological or tract neoplasm, urinary or tract neoplasms, urinary or urinary tract cancer or urinary tract cancers or urinary tract neoplasm or urinary tract neoplasms or urologic cancer or urologic cancers or urologic neoplasm or urologic neoplasms or urological cancer or urological cancers or urological neoplasm or urological neoplasms or (bladder cancer or bladder cancers or bladder neoplasm or bladder neoplasms or bladder tumour or bladder tumours or cancer, bladder or cancer, urinary bladder or “cancer of bladder” or “cancer of the bladder” or “malignant tumour of urinary bladder” or neoplasm, bladder or neoplasm, urinary bladder or neoplasms, bladder or tumour, bladder or tumours, bladder or urinary bladder cancer or urinary bladder neoplasm or urinary bladder neoplasms) or (cancer, urethra or cancer, urethral or “cancer of urethra” or “cancer of the urethra” or cancers, urethra or cancers, urethral or neoplasm, urethra or neoplasm, urethral or neoplasms, urethra or neoplasms, urethral or urethra cancer or urethra cancers or urethra neoplasm or urethra neoplasms or urethral cancer or urethral cancers or urethral neoplasm or urethral neoplasms) or (cancer, ureteral or “cancer of ureter” or “cancer of the ureter” or cancers, ureteral or neoplasm, ureteral or neoplasms, ureteral or “neoplasms of ureter” or ureter cancer or “ureter, cancer of” or ureter cancers or ureter neoplasm or ureter neoplasms or ureteral cancer or ureteral cancers or ureteral neoplasm or ureteral neoplasms) or (cancer, kidney or cancer, renal or “cancer of kidney” or “cancer of the kidney” or cancers, kidney or cancers, renal or kidney cancer or kidney cancers or kidney neoplasm or kidney neoplasms or neoplasm, kidney or neoplasm, renal or neoplasms, kidney or neoplasms, renal or renal cancer or renal cancers or renal neoplasm or renal neoplasms)).mp.
2. Patient population	exp Cystectomy/ or exp Nephrectomy/or (cystectomies or cystectomy or cystectomy, partial or cystectomy, radical or partial cystectomies or partial cystectomy or radical cystectomies or radical cystectomy or heminephrectomies or heminephrectomy or nephrectomies or nephrectomy).mp.
Algorithm	#1 AND #2
3. Outcome	exp Patient Reported Outcome Measures/or Patient Outcome Assessment/exp Self Report/ “Surveys and Questionnaires”/ or “Quality of Life”/or (Quality of life or QOL or hrql or QOL).ab,ti. (Questionnaire* or Scale* or Instrument* or diaries or diary or Survey* or patient‐report* or self‐administ* or self‐report* or assessment* or Tool* or Measure* or Index or Inventory or checklist* or Patient‐reported outcome* or PROMS).ab,ti. (interviewer‐administ* or clinician‐administ* or clinician‐rat* or clinician‐report* or physician‐administ* or physician‐rat* or physician‐report*).ab,ti. (Caregiver* or proxy or proxies or carer* or caregiver‐administ* or carer‐administered or Proxy‐administ* or Parent* or spouses* or family or families or partner*).ab,ti.
4. Intervention	exp. adaptive clinical trial or clinical study or clinical trial, all or clinical trial, phase i or clinical trial, phase ii or clinical trial, phase iii or clinical trial, phase iv or clinical trial protocol or clinical trial or comparative study or controlled clinical trial or equivalence trial or multicenter study or observational study or pragmatic clinical trial or randomized controlled trial or validation study
5. Timeframe limit	2014‐June 2024
Algorithm	[Urologic cancer terms] AND [cystectomy terms] AND [COA + QOL terms]; limited to clinical studies with an English abstract published
# hits	**142**
5. Experts review of title and abstract	Excluded: Not assessing the intervention of interest (n = 69) Out of scope of interest (n = 46) NMIBC not specified (n = 8) Population (n = 5) Study protocol only (n = 3) Duplicate (n = 1) Study not assessing the time frame of interest (n = 1) Publication type (n = 1) No statistical significance in group of interest (n = 1)
# selected	**7**

Source: Medline database (via OVID platform).

**TABLE A2:** Search strategy for the retrieval of articles for qualitative studies.

Search #	Algorithm
1. Condition terms	exp Urologic Neoplasms/or (cancer, urinary tract or cancer, urologic or cancer, urological or “cancer of urinary tract” or “cancer of the urinary tract” or cancers, urinary tract or cancers, urologic or cancers, urological or neoplasm, urinary tract or neoplasm, urologic or neoplasm, urological or neoplasms, urinary tract or neoplasms, urologic or neoplasms, urological or tract neoplasm, urinary or tract neoplasms, urinary or urinary tract cancer or urinary tract cancers or urinary tract neoplasm or urinary tract neoplasms or urologic cancer or urologic cancers or urologic neoplasm or urologic neoplasms or urological cancer or urological cancers or urological neoplasm or urological neoplasms or (bladder cancer or bladder cancers or bladder neoplasm or bladder neoplasms or bladder tumour or bladder tumours or cancer, bladder or cancer, urinary bladder or “cancer of bladder” or “cancer of the bladder” or “malignant tumour of urinary bladder” or neoplasm, bladder or neoplasm, urinary bladder or neoplasms, bladder or tumour, bladder or tumours, bladder or urinary bladder cancer or urinary bladder neoplasm or urinary bladder neoplasms) or (cancer, urethra or cancer, urethral or “cancer of urethra” or “cancer of the urethra” or cancers, urethra or cancers, urethral or neoplasm, urethra or neoplasm, urethral or neoplasms, urethra or neoplasms, urethral or urethra cancer or urethra cancers or urethra neoplasm or urethra neoplasms or urethral cancer or urethral cancers or urethral neoplasm or urethral neoplasms) or (cancer, ureteral or “cancer of ureter” or “cancer of the ureter” or cancers, ureteral or neoplasm, ureteral or neoplasms, ureteral or “neoplasms of ureter” or ureter cancer or “ureter, cancer of” or ureter cancers or ureter neoplasm or ureter neoplasms or ureteral cancer or ureteral cancers or ureteral neoplasm or ureteral neoplasms) or (cancer, kidney or cancer, renal or “cancer of kidney” or “cancer of the kidney” or cancers, kidney or cancers, renal or kidney cancer or kidney cancers or kidney neoplasm or kidney neoplasms or neoplasm, kidney or neoplasm, renal or neoplasms, kidney or neoplasms, renal or renal cancer or renal cancers or renal neoplasm or renal neoplasms)).mp.
2. Intervention terms	exp Cystectomy/ or exp Nephrectomy/or (cystectomies or cystectomy or cystectomy, partial or cystectomy, radical or partial cystectomies or partial cystectomy or radical cystectomies or radical cystectomy or heminephrectomies or heminephrectomy or nephrectomies or nephrectomy).mp.
3. Qualitative terms	exp Qualitative research/ or exp Focus Groups/ or exp Interviews as topic/ or exp Narration/ or exp “Personal Narratives as Topic”/or (Semi‐structured interview* or semistructured interview* or structured interview* or in‐depth interview* or indepth interview* or Qualitative interview* or exploratory interview* or individual interview* or “face‐to‐face” or focus group* or qualitative stud* or qualitative research).mp.
Algorithm	#1 AND #2 AND #3
4. Timeframe limit	2013–2022
# hits	**50**
5. Experts review of title and abstract	Excluded: Out of concept of interest (n = 20) No qualitative methods (n = 8) Population (n = 6) Publication type (n = 2) Duplicate (n = 1) Language (n = 1) No abstract (n = 1) Nephrectomy (n = 2)
# selected	**11**

Source: Medline database (via OVID platform).
